# The risk and latency evaluation of secondary primary malignancies of cervical cancer patients who received radiotherapy: A study based on the SEER database

**DOI:** 10.3389/fonc.2022.1054436

**Published:** 2023-01-19

**Authors:** Mengjie Chen, Xinbin Pan, He Wang, Desheng Yao

**Affiliations:** ^1^ The Department of Gynecological Oncology, Guangxi Medical University Cancer Hospital, Nanning, Guangxi, China; ^2^ The Department of Radiotherapy, Guangxi Medical University Cancer Hospital, Nanning, Guangxi, China

**Keywords:** secondary primary malignancies, cervical cancer, radiotherapy, nomogram, SEER

## Abstract

**Objectives:**

To study the risk factors for the onset of secondary primary malignancies (SPM) and the latency between SPM and cervical cancer after radiotherapy.

**Methods:**

We selected patients with cervical cancer who underwent radiotherapy between 2000 and 2019 from the Surveillance, Epidemiology, and End Results (SEER) database. And the data of patients with cervical cancer who underwent radiotherapy in Guangxi Medical University Cancer Hospital during January 1,1997 to December 31,2016 were collected and analyzed. The factors associated with SPM onset and latency were then estimated by nomograms based on logistic regression and a complete risk model. Dynamic risk plots were performed by Poisson regression.

**Results:**

A total of 32,313 cases of cervical cancer who underwent radiotherapy were downloaded from the SEER database; of these, 19,439 cases had a complete dataset and were included in the final analysis. In total, 561 cases suffered from SPM; the remaining 18,878 did not. And a total of 1486 cases of cervical cancer who underwent radiotherapy from Guangxi Medical University Cancer Hospital were analyzed, 27 cases caught SPM and the rest of 1459 cases did not. Patients with SPM were older than those without SPM(p=0.000); significant associations were also identified between SPM and white race(p=0.000), localized stage (p=0.000), squamous carcinoma (SCC)(p=0.003), surgery(p=0.000), and combination radiotherapy (p=0.026). A logistic regression nomogram showed that older age (HR:1.015, 95%CI:1.009-1.021, p=0.000), localized stage (HR:4.056, 95%CI: 2.625-6.269, p=0.000) and regional stage (HR: 3.181, 95%CI:2.094-4.834, p=0.000), white (HR: 1.722, 95%CI:1.145-2.590, p=0.000) and black race (HR: 1.889, 95%CI:1.327-2.689, p=0.000), and the receipt of surgery (HR: 1.381, 95%CI:1.151-1.657, p=0.000) were all independent risk factors for the onset of SPM. The largest proportion of cases involved SPM in the female reproductive system. A dynamic risk plot showed that age, race, stage, and surgery had impacts on the latency of SPM onset. A competing risk regression analysis nomogram showed that age (HR: 1.564, 95%CI: 1.272-1.920, p=0.000), surgery (HR: 1.415, 95%CI: 1.140-1.760, p=0.002), localized stage (HR: 8.035, 95%CI: 4.502-14.340, p=0.000) and regional stage (HR: 4.904, 95%CI: 2.790-8.620, p=0.000), and black race (HR: 1.786, 95%CI: 1.161-2.750, p=0.008) all had significant impacts on the cumulative incidence and latency of SPM.

**Conclusions:**

Advanced age, the receipt of surgery, earlier stages, and white and black race were identified as risk factors for SPM onset and influenced latency in patients with cervical cancer after radiotherapy.

## Introduction

Cervical cancer is the fourth most frequently diagnosed form of cancer and the fourth leading cause of death by cancer in women, with an estimated 604,000 new cases and 342,000 deaths worldwide in 2020 (1). Surgeries, radiotherapy, and chemotherapy are the classical and most effective treatments for cervical cancer (2). Radiotherapy is considered as an indispensable treatment for cervical cancer, especially for patients with advanced stages of cervical cancer; this form of treatment is almost equivalent to surgery with regard to controlling tumor progression.

A previous study reported that cervical cancer patients who underwent radiotherapy are vulnerable to secondary primary cancer of organs in the pelvis (3). However, radiotherapy is associated with many side effects including secondary cancer (4). As the main target for the biological effects of ionizing radiation, the treatment effects on DNA can be categorized in three groups: genetic effects, epigenetic effects, and bystander effects (5). Generally, radiotherapy kills tumor cells by damaging DNA. However, this form of radical injury can involve the adjacent organs and can change the genome of normal cells, thus creating a latent danger for secondary cancer.

Although many cervical cancer patients receive radiotherapy, not all of them suffer from secondary cancer. There are likely to be other factors that influence the final outcomes. Therefore, in this study, we analyzed patients with cervical cancer receiving radiotherapy and suffering from secondary cancer in order to demonstrate risk factors for the onset of secondary cancer.

## Methods

### Patient selection

Female patients diagnosed with cervical cancer as the first primary cancer were identified from 17 registries of the Surveillance, Epidemiology, and End Results (SEER) database between the January 1, 2000 and the December 31, 2019. All primary cancer sites were coded according to the International Classification of Diseases for Oncology, Third Edition. The included patients were pathologically diagnosed with endocervix cancer (C53.0), exocervix cancer (C53.1), overlapping lesions of cervix uteri cancer (C53.8), and cervix uteri cancer (C53.9). The exclusion criteria included patients in whom rectal cancer was not the first primary cancer, patients who were aged younger than 20 years, patients who survived less than 5 years after the diagnosis of rectal cancer, patients with non-epithelial tumors, patients who did not undergo radiotherapy, and patients with missing data relating to radiotherapy, surgery, age, tumor stage, race, survival status, or follow-up. This study was approved by the Ethics Committee of Guangxi Medical University Cancer Hospital. Access to and the use of SEER data did not require informed patient consent. This study followed the Strengthening the Reporting of Observational Studies in Epidemiology (STROBE) reporting guideline for cohort studies. The study was conducted in accordance with the Declaration of Helsinki (as revised in 2013).

Demographic and clinical variables were collected from the SEER database. We included patients with cervical cancer who received radiotherapy. Then, 70% of the cases included from the SEER database were randomly selected as a training set; the remaining patients were used as an internal validation set. A range of data were collated for each patient, including age, race, pathological type, histological grade, stage, surgery, chemotherapy, radiotherapy, year of diagnosis, overall survival (OS), and survival status.

### External test cohort

The clinical data of 1486 cases diagnosed with cervical cancer as the first primary cancer who admitted in Guangxi Medical University Cancer Hospital during January 1,1997 to December 31,2016 were collected and analyzed, including age, race, pathological type, histological grade, stage, surgery, chemotherapy, radiotherapy, year of diagnosis, overall survival (OS), and survival status. This study was approved by the Ethics Committee of Guangxi Medical University Cancer Hospital.

### Definition and follow-up of SPM

**Figure 1:**
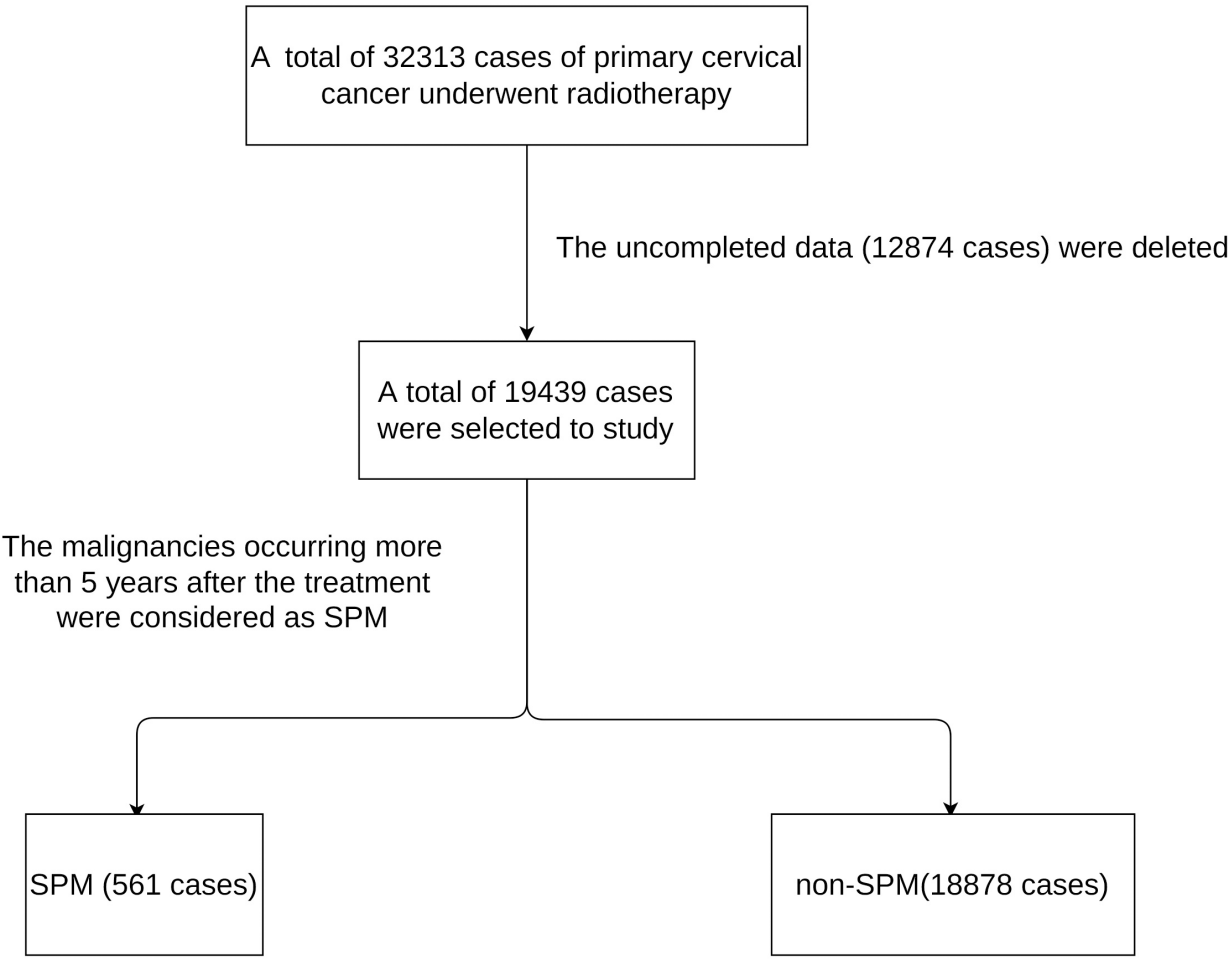
The flow chart of patients selection.

The primary outcome of this study was the development of SPM, which was defined as any type of SPM occurring more than 5 years after the treatment of cervical cancer because of at least a 5-year latency period from radiotherapy exposure to the occurrence of a solid tumor. The SEER database adheres to the International Classification of Diseases for Oncology (Third Edition) guidelines and distinguishes SPM from recurrent disease. To obtain comprehensive estimations for the risk of SPM, the risk for all types of SPM was estimated (expect for tumors in the male reproductive system). The follow-up for SPM began 5 years after the diagnosis of cervical cancer and ended at the date of diagnosis of any SPM. The cut-off point for follow-up was defined as January 1, 2016 (April 2019 SEER data release). A flow chart showing the experimental design is shown in **Figure 1**.

### Statistical analysis

All statistical analyses were performed by R 4.1.0 and Rstudio software. Continuous parametric and normal distributed data were compared using t-tests or rank-sum tests while categorical data were compared using the chi-squared test. Fisher exact test was used for categorical data when frequencies were below 5. The Mann-Whitney test was used to analyze continuous variables with a non-normal distribution, respectively. Univariable and multivariable logistical regression models were performed for the training cohort using the “rms,” “Hmisc,” and “lattice” packages in R. Nomograms were generated by the “regplot” in R to predict the risk of secondary cancer onset. The performance of the nomogram was further validated using the test cohort. The discrimination accuracy of the nomogram was quantified using Harrell’s concordance index (the C-index). Furthermore, a calibration curve with 400 resamples from bootstrapping was used to assess the nomograms. The “pROC” package in R was used to plot calibration curves and receiver operating characteristic curves (ROCs) to assess the calibration of the nomogram. The clinical utility of the nomogram was evaluated using decision curve analysis (DCA) which was performed in the “rmda” package in R.

The radiotherapy-associated risk (RR) was estimated by Poisson regression analysis with the relative risk and 95% confidence intervals (CIs) of SPM development for patients with cervical cancer who received radiotherapy. To further evaluate the dynamic risks and incidence of SPM associated with different risk factors, we analyzed the RRs when stratified by latency time since SPM diagnosis in patients with cervical cancer. The data used for Poisson regression analysis were evaluated by the overdispersion test in the “qcc” package in R.

Fine-Gray competing risk regression analysis was used to assess the cumulative incidence of SPM development. Experiencing a non-SPM and dying of all causes were considered to be competing events when we calculated hazard ratios (HRs) and 95% CIs for SPM occurrence. A multivariable risk model was built by a backward selection procedure with variables and a two-sided P < 0.05 in univariable analyses; significant factors were then included in multivariable analyses. Multivariable analysis was conducted by weighting the formula by the “crprep” function in R. These analyses were completed by the “mstate” and “cmprsk” packages in R. Calibration curves and ROCs were used to assess the performance of the nomogram.

## Results

### Characteristics of patient from SEER

A total of 32,313 cases of primary cervical cancer met the selection criteria (**Supplement 1**). Non-complete data were deleted and finally a total of 19,439 cases of cervical cancer cases were included from 2000 to 2019. In total, 561 cases suffered from SPM; the remaining 18,878 cases did not. The demographic and clinicopathological variables of the entire SEER cohort are listed in **Supplement 2**.

**Figure 2:**
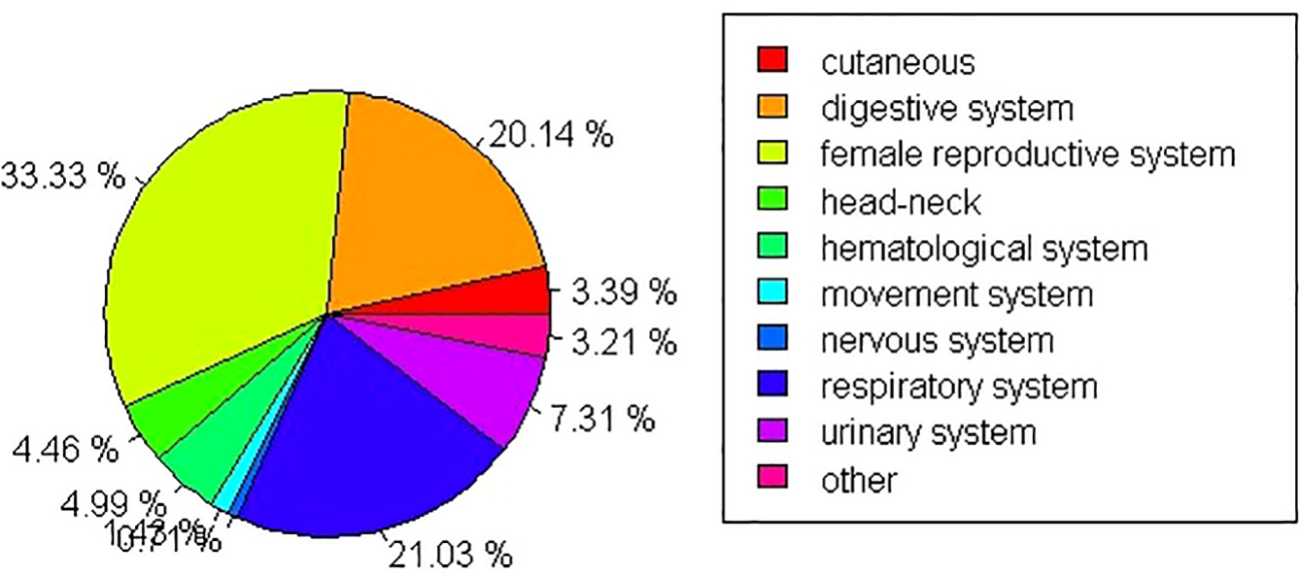
The percentages of SPM onset sites.

With regard to SPM sites, secondary female reproductive cancers (including ovary, corpora uteri, vulvar, vaginal, and breast cancers) showed the highest incidence in SPM (33.33%) followed by cancers of the respiratory system (21.03%). The lowest proportion of SPM cases involved nervous system tumors (0.71%). Further details are shown in **Figure 2**.

### Characteristics of patient from external test data

A total of 1486 cases diagnosed with cervical cancer and receiving radiotherapy were analyzed (**Supplement 3**). There were 27 cases suffering from SPM and the rest of 1459 cases were not. The race of all patients was yellow race. The demographic and clinicopathological variables of the entire SEER cohort are listed in **Supplement 4**.

As to the sites of SPM, 8 cases were diagnosed with primary lung cancer, which was the most common SPM in the external test cohort, accounting for 29.63%. The following was primary bladder cancer, breast cancer, vulva cancer, rectal cancer and nasopharyngeal cancer, taking up 7.41% (2 cases) of all SPM, respectively. And the remain SPM included 1 case (3.70%) of primary ovarian cancer, carcinoma of head of pancreas, urethra cancer, gastric cancer, hepatic cancer, parotid gland cancer, thyroid cancer and synovial sarcoma of nervi ischiadicus and iliopsoas, respectively.

### Development and validation of nomograms of predicting secondary cancer occurrence

**Figure 3:**
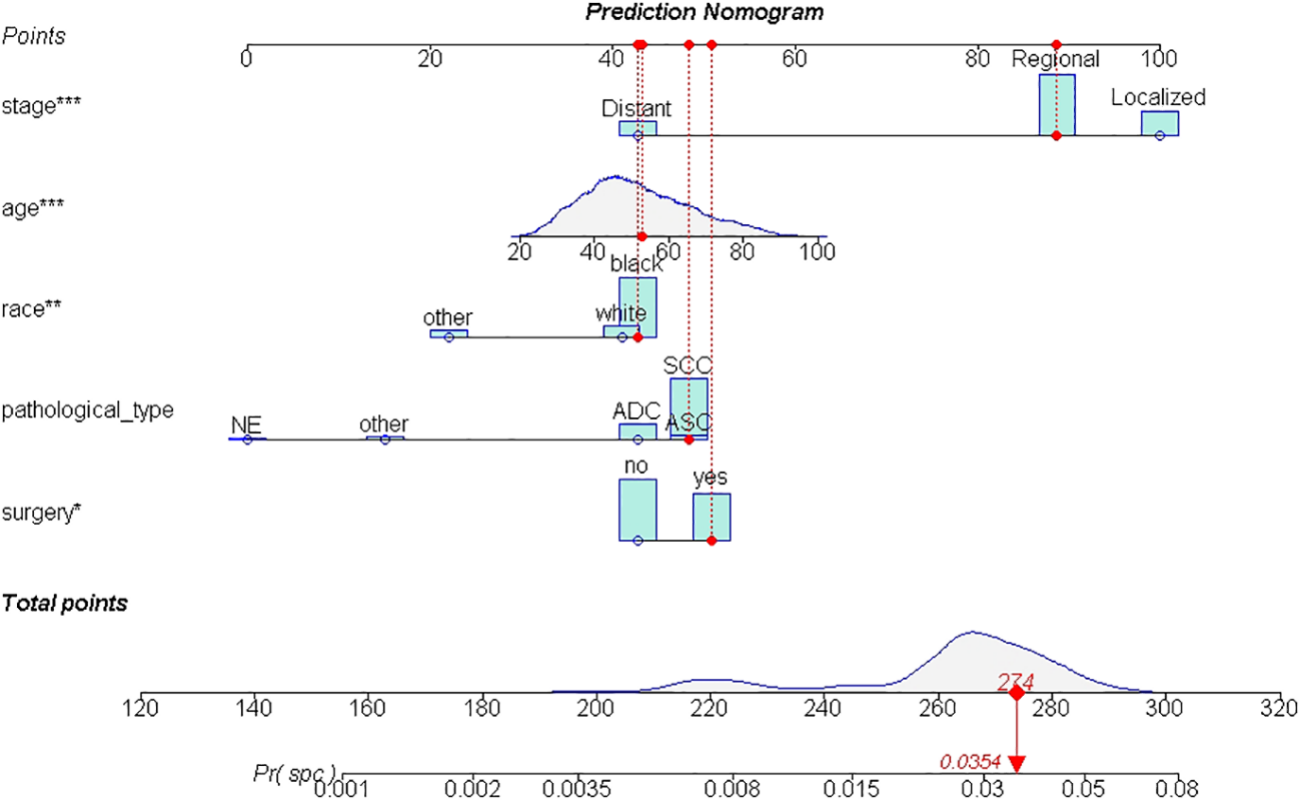
The nomogram of SPM risk prediction. *: p value <0.010; **: p value <0.005; ***: p value <0.000.

**Figure 4:**
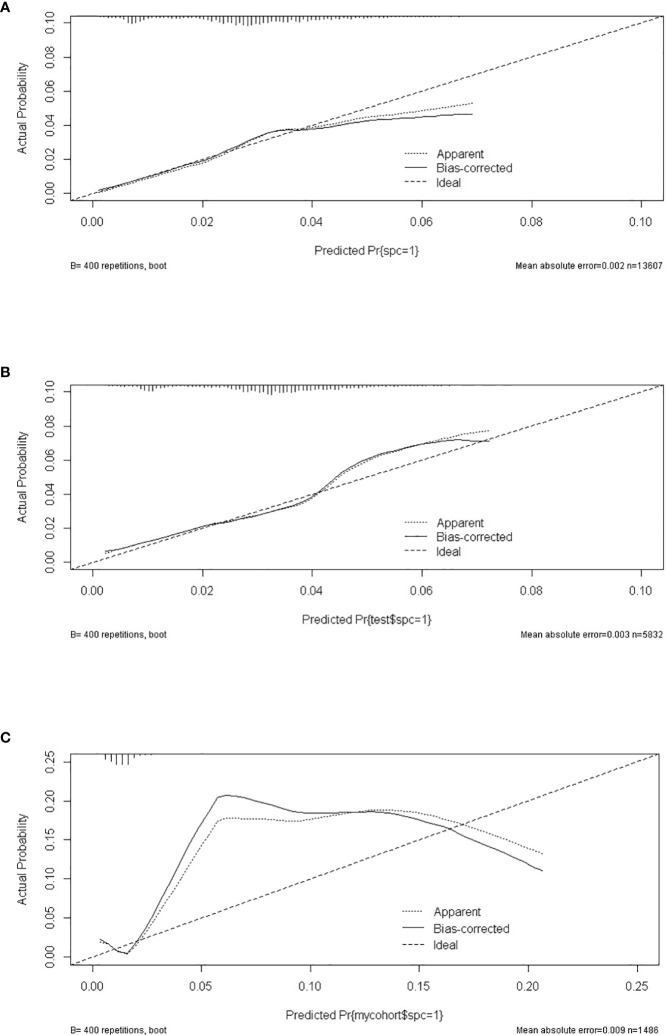
The calibrate curves of SPM risk prediction nomogram. **(A)** trainset; **(B)** testset; **(C)** external test cohort.

**Figure 5:**
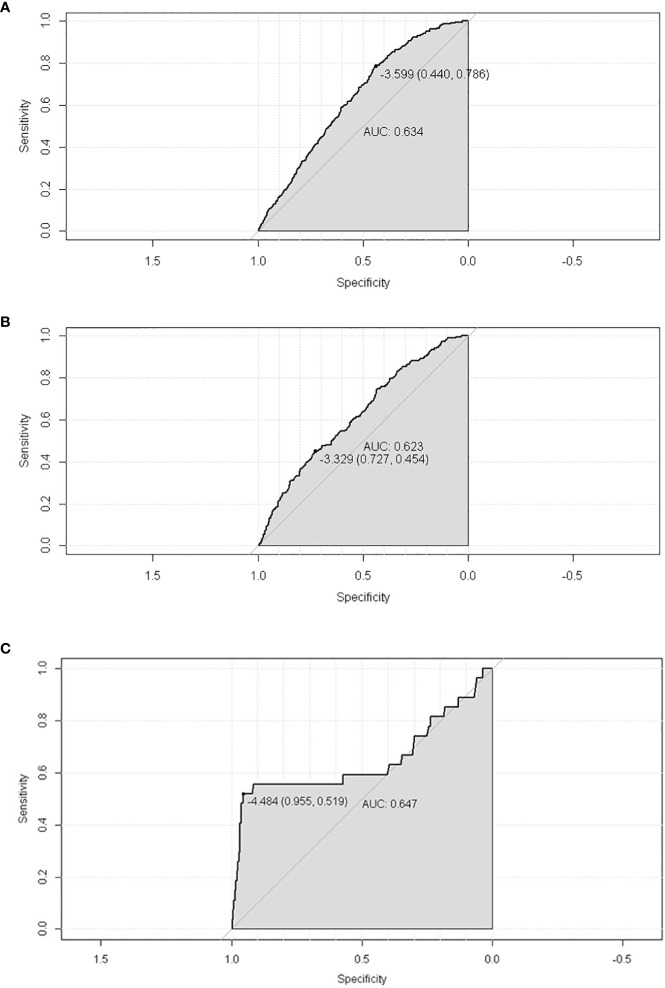
The ROC of SPM risk prediction nomogram. **(A)** trainset; **(B)** testset; **(C)** external test cohort.

A total of 19,439 cases were randomly divided into a training set and a test set in a ratio of 7:3. Detailed results of the univariable and multivariable logistic regression analysis of predicative variables from the training cohort are summarized in **Table 1**. Advanced age, surgery, earlier stages, SCC (squamous carcinoma), and white and black race were identified as independent risk factors for secondary cancer following cervical cancer the details of scores listed in **Supplement 5**. Then, we established a nomogram based on the results of multiple logistic regression analysis to screen for the risk factors of secondary cancer occurrence (**Figure 3**). The calibration curves showed fair agreements between the predicted and actual observations in the training cohort (**Figure 4A**). The C-index and AUC for the training cohort was 0.635 and 0.634, respectively (**Figure 5A**).

**Table 1:** Univariable and multivariable analysis of the risk factors of secondary cancer.

	Univariable analysis			Multivariable analysis		
	HR	95% CI	P	HR	95% CI	P
**Age**	1.011	1.005-1.017	**0.000**	1.015	1.009-1.021	**0.000**
**Races(other)**			**0.003**			**0.002**
white	1.652	1.102-2.478	**0.015**	1.722	1.145-2.590	**0.009**
black	1.820	1.280-2.588	**0.001**	1.889	1.327-2.689	**0.000**
**Pathological types(other)**			**0.026**			**0.022**
SCC	3.385	1.259-9.101	**0.016**	2.974	1.104-8.013	**0.031**
ADC	2.974	1.088-8.129	**0.034**	2.359	0.860-6.476	0.096
ASC	3.032	1.045-8.793	**0.041**	2.654	0.911-7.731	0.074
NE	0.440	0.049-3.957	0.464	0.485	0.054-4.375	0.519
**Grades(undifferentiated)**			0.113	/	/	/
High differentiation	2.109	1.088-4.088	0.027	/	/	/
Medium differentiation	1.902	1.035-3.498	0.039	/	/	/
Low differentiation	1.732	0.942-3.183	0.077	/	/	/
**Stages(distant)**			**0.000**			**0.000**
Localized	4.728	3.083-7.249	**0.000**	4.056	2.625-6.269	**0.000**
Regional	3.464	2.286-5.249	**0.000**	3.181	2.094-4.834	**0.000**
**Radiotherapy(other)**			**0.028**			0.057
Beam radiotherapy	0.503	0.271-0.935	**0.030**	0.499	0.268-0.931	0.029
Brachytherapy	0.670	0.341-1.316	0.245	0.628	0.318-1.238	0.179
Combination	0.612	0.330-1.137	0.120	0.596	0.320-1.113	0.104
Radioisotopes	1.145	0.308-4.259	0.839	1.046	0.278-3.930	0.947
**Surgery**	1.185	1.013-1.386	**0.034**	1.381	1.151-1.657	**0.001**
**Chemotherapy**	1.152	0.949-1.398	0.153	/	/	/

The p values less than 0.05 were highlighted with bold values.

**Figure 6:**
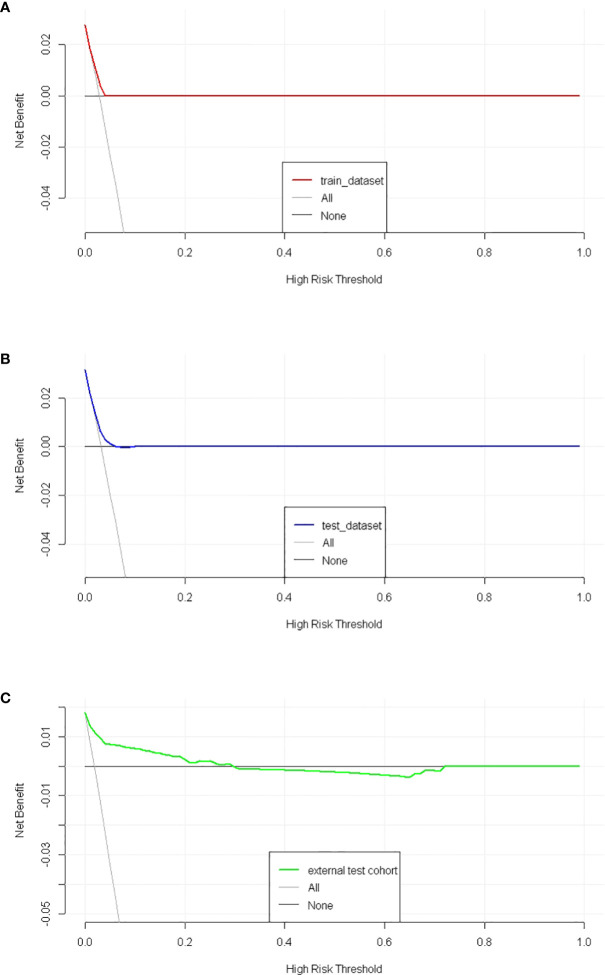
The decision curves of SPM risk prediction nomogram. **(A)** trainset; **(B)** testset; **(C)** external test cohort.

The discriminatory ability of these nomograms was further validated in the testing set. The calibration curves and ROC curves showed fair consistency between actual probability and nomogram-predicted probability in the testing set (**Figures 4B**, **5B**). Moreover, to verify the model further, the external test cohort was used to test. Due to single race in external test cohort, the calibration curve was not perfect enough (**Figure 4C**). The C-index and AUC for external test cohort were the 0.647 and o.647, respectively, which was approximate to that of training set (**Figure 5C**). The DCA demonstrated the clinical benefits of the nomogram predictions (**Figures 6A–C**).

### Dynamic risk and incidence evaluation for secondary primary malignancies

**Figure 7:**
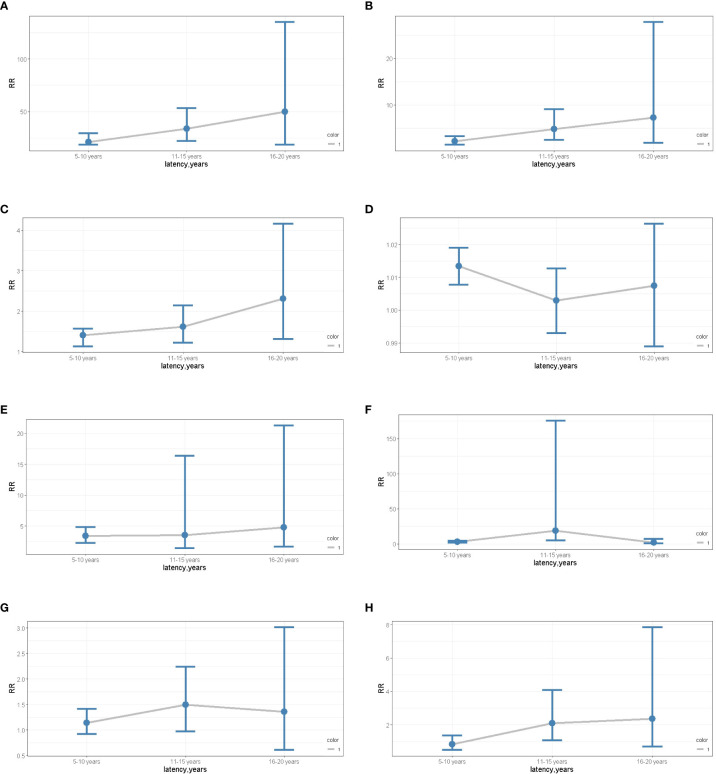
**(A)** The dynamic RR plots of White races (compared to the black). **(B)** the dynamic RR plots of other races (compared to the black); **(C)** the dynamic RR plots of receiving surgery (compared to non-surgery patients); **(D)** the dynamic RR plots of age older than 50 years old (compared to age younger than 50 years old patients); **(E)** the dynamic RR plots of localized stage (compared to distant metastases); **(F)** the dynamic RR plots of regional stage (compared to distant metastases); **(G)** the dynamic RR plots of SCC (compared to the ADC); **(H)** the dynamic RR plots of ASC (compared to the ADC).

To assess the risks of SPM in different latency periods, ages, stages, pathological types, and surgery, we used Poisson regression to evaluate dynamic risk according to the nomogram results. Analysis showed that race was a risk factor for SPM. Compared to black females, the risk of SPM in white females showed an upward trend over 5-15 years but fell in late latency. The risk of SPM in females of other races increased during early and late latency (**Figures 7A, B**). Similarly, an increased risk of SPM was observed in patients receiving surgeries (**Figure 7C**). Interestingly, an advanced age was only associated with an increased risk during early latency (**Figure 7D**). Furthermore, the risk of localized and regional stages presented an increased tendency over 5-10 years and 11-15 years of latency, although there was limited effect during late latency (**Figures 7E, F**). However, the pathological type of primary cervical cancer had no impact on SPM onset despite latency (**Figures 7G, H**). Further details are shown in **Table 2**.

**Table 2:** The dynamic risk in different period latency.

	Latency	RR	95% CI	P
**Age>50 years old**	**5-10 years**	1.01	1.01-1.02	**0.000**
	**11-15years**	1.00	0.99-1.10	0.623
	**16-20years**	1.01	0.99-1.03	0.511
**Race-white**	**5-10 years**	21.06	18.66-29.46	**0.000**
	**11-15years**	33.56	22.12-53.39	**0.000**
	**16-20years**	49.94	18.46-135.07	**0.000**
**Race-other**	**5-10 years**	2.22	1.44-3.31	**0.000**
	**11-15years**	4.82	2.49-9.08	**0.000**
	**16-20years**	7.25	1.90-27.75	**0.020**
**Stage-localized**	**5-10 years**	34.02	2.23-4.84	**0.000**
	**11-15years**	3.49	1.39-16.36	**0.000**
	**16-20years**	4.77	1.64-21.27	**0.040**
**Stage-regional**	**5-10 years**	3.01	2.13-4.41	**0.000**
	**11-15years**	18.72	5.08-175.47	**0.000**
	**16-20years**	2.13	0.63-7.27	0.310
**Surgery-Yes**	**5-10 years**	1.41	1.13-1.57	**0.000**
	**11-15years**	1.62	1.22-2.15	**0.010**
	**16-20years**	2.30	1.32-4.16	**0.020**
				
**Pathological type-SCC**	**5-10 years**	1.14	0.93-1.41	0.310
	**11-15years**	1.49	0.97-2.24	0.110
	**16-20years**	1.36	0.61-3.01	0.530
**Pathological type-ASC**	**5-10 years**	0.85	0.50-1.36	0.590
	**11-15years**	2.10	1.08-4.08	0.070
	**16-20years**	2.36	0.71-7.84	0.240
**Pathological type-NE**	**5-10 years**	0.21	0.02-0.77	0.120
	**11-15years**	0.00	0.00-Inf	0.980
	**16-20years**	0.00	0.00-Inf	0.990
**Pathological type-other**	**5-10 years**	0.40	0.11-0.79	0.110
	**11-15years**	0.44	0.13-0.91	0.420
	**16-20years**	0.00	0.00-Inf	0.990

The p values less than 0.05 were highlighted with bold values.

### Cumulative incidences of SPM

**Figure 8:**
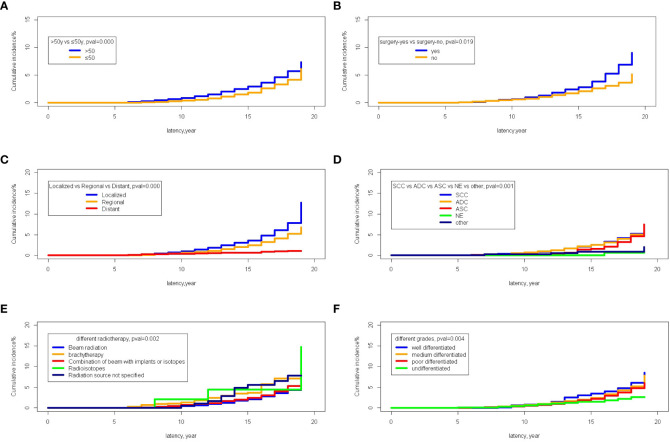
The comparison of cumulative incidences of SPM. **(A)** The comparison between age older than 50 years old and younger than 50 years old; **(B)** The comparison between the patients receiving surgery or not; **(C)** The comparison among different stages; **(D)** The comparison among different pathological types of primary cervical cancer; **(E)** The comparison among different methods of radiotherapies; **(F)** The comparison among different histological grades of primary cervical cancer.

The Fine-Gray test showed that the incidence of the patients older than 50 years was 2.91% while that of patients younger than 50 was 1.87% (P=0.000). The incidence of SPM was 2.08% in patients with no surgery and 2.81% in patients receiving surgery (P=0.000). In stage-specific analyses, the cumulative incidence of localized and regional stages was significantly higher in patients with metastases (3.57% and 2.47% vs 0.61%; P=0.000). Different pathological types of cervical cancer showed significantly different cumulative incidences of SPM; the cumulative incidences of SCC (squamous cells carcinoma, SCC), ADC (adenocarcinoma, ADC), ASC (adenosquamous carcinoma, ASC), and NE (neuroendocrine carcinoma, NE) and others were 2.50%, 2.54%, 1.61%, 0%, and 0.90% (P=0.000). Moreover, different types of radiotherapy exerted differential impacts on the cumulative incidence of SPM; the incidences of beam radiation, brachytherapy, a combination of beam radiation with implants or isotopes, radioisotopes, and a non-specified source of radiation were 2.07%, 3.70%, 2.47%, 4.47%, and 5.57%, respectively (P=0.002). Furthermore, there was a significantly decreasing trend of cumulative incidence with poorer histological grades; the cumulative incidences of well, medium, poor differentiated, and undifferentiated cervical cancer were 3.53%, 2.48%, 2.22%, and 1.53%, respectively (P=0.004) (**Figures 8A–F**).

**Figure 9:**
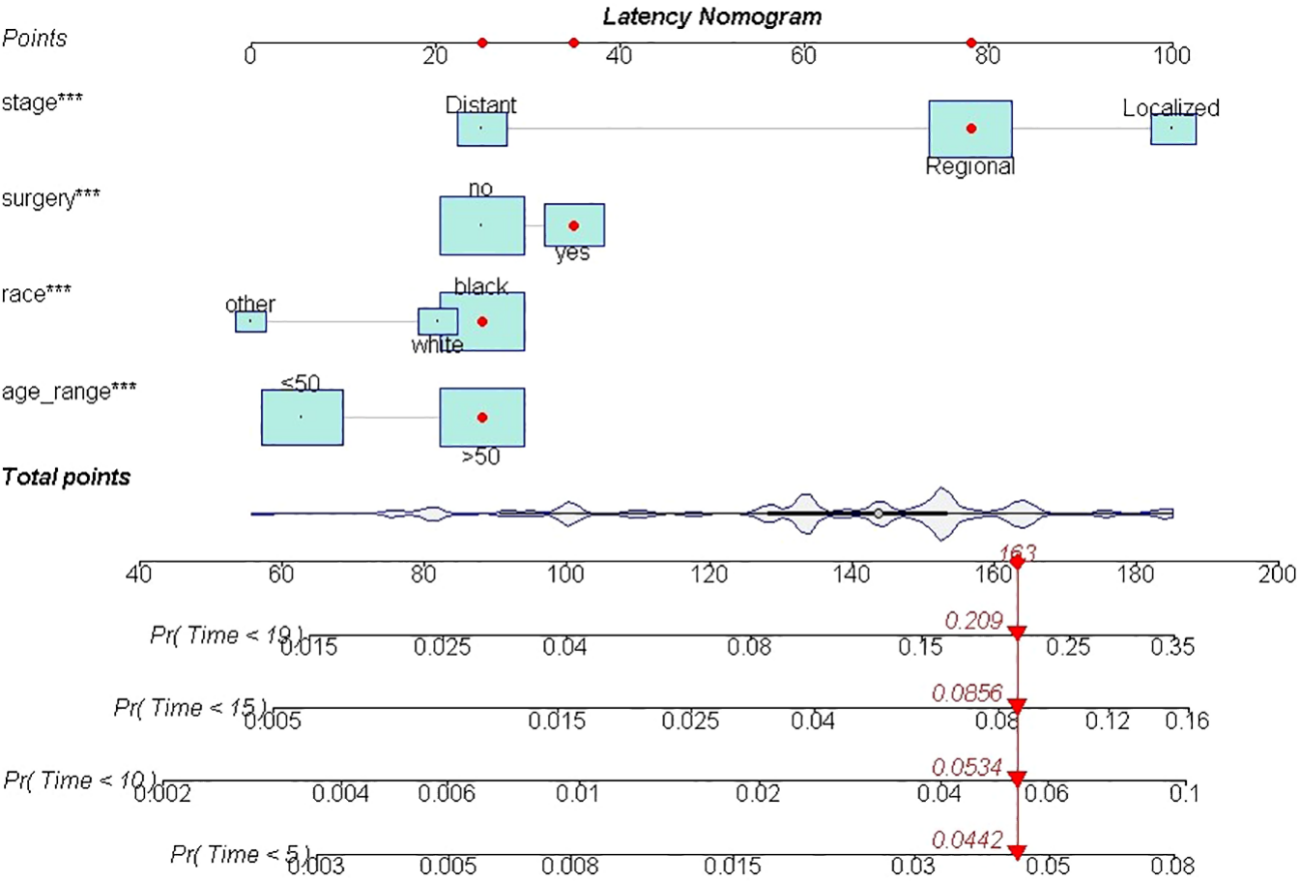
The nomogram of predicting the latency between cervical cancer and SPM. ***, It meant pvalue less than 0.000.

**Figure 10:**
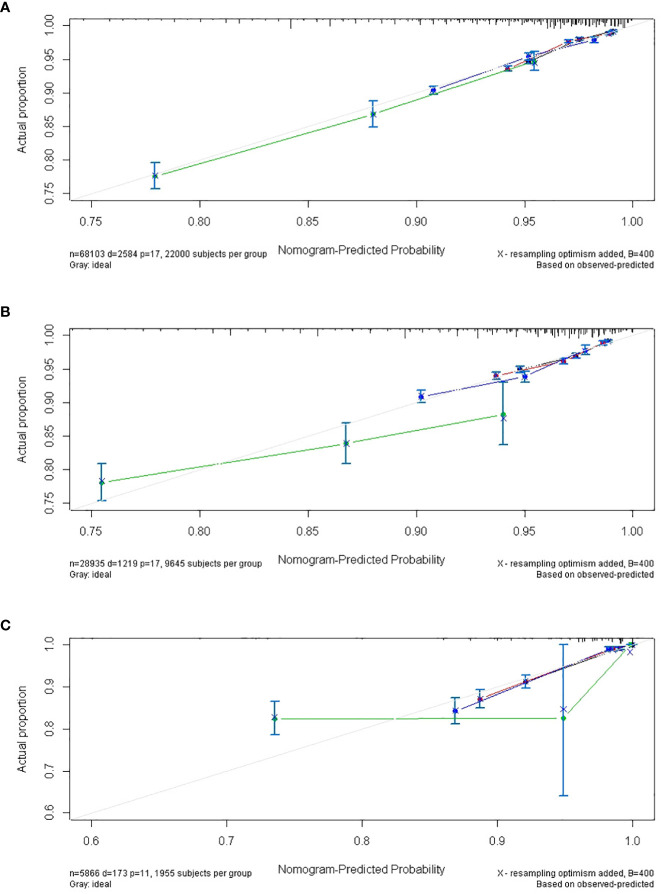
The calibrate curves of the nomogram of predicting the latency between cervical cancer and SPM. **(A)** trainset; **(B)** testset; **(C)** external test cohort. (black line: 5-year calibrate curve; red line: 10-year calibrate curve; blue line: 15-year calibrate curve; green line: 19-year calibrate line).

**Figure 11:**
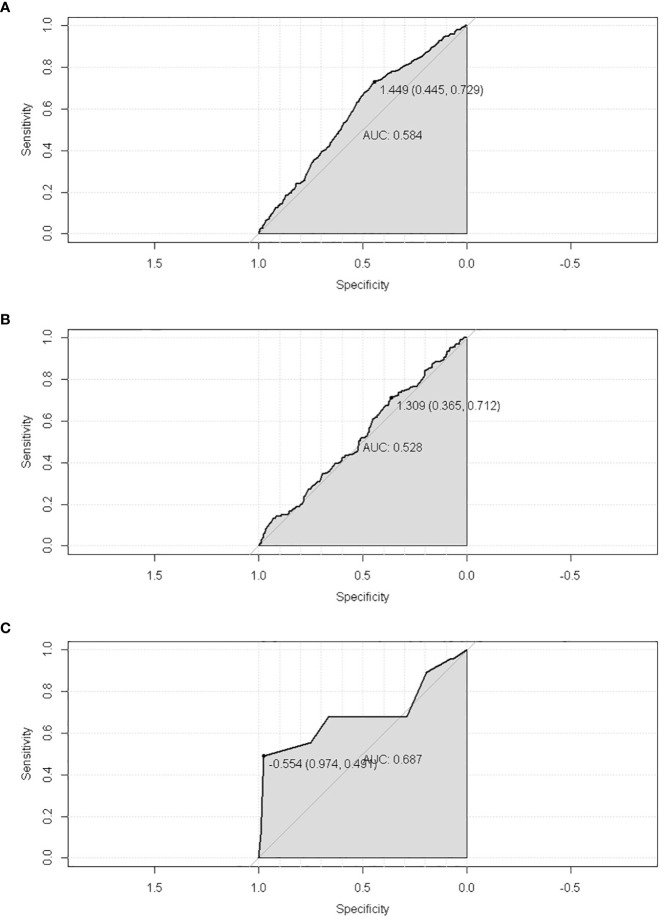
The ROC of the nomogram of predicting the latency between cervical cancer and SPM. **(A)** trainset; **(B)** testset; **(C)** external test cohort.

Furthermore, multiple cumulative incidences were analyzed based on age, surgery, stages, radiotherapy, grades, and pathological types. We found that age, surgery, stage, and race had significant impacts on the cumulative incidence of SPM. Further details are shown in **Table 3**. Moreover, these data were weighted and randomly divided into a training set and a testing set at a ratio of 7:3. We also established a nomogram for multiple cumulative incidences to predict the latency of SPM onset (**Figure 9**), the details of scores listed in **Supplement 6**. The C-indexes for the training cohort and testing cohort were 0.715 and 0.692, respectively. The discrimination ability of these nomograms was further validated in the testing set. Calibration curves and ROC curves showed fair consistency between the actual probability and the nomogram-predicted probability in the testing set (**Figures 10A, B**, **11A, B**).

**Table 3:** The multiple competes risk model.

	RR	95% CI	P
**>50 years old**	1.564	1.272-1.920	**0.000**
Races(other)			
white	1.566	0.954-2.570	0.076
black	1.786	1.161-2.750	**0.008**
Pathological types (other)			
SCC	2.707	0.867-8.450	0.086
ADC	2.298	0.720-7.330	0.160
ASC	2.719	0.794-9.310	0.110
NE	0.622	0.064-6.080	0.680
Grades(undifferentiated)			
High differentiation	2.291	0.745-3.850	0.061
Medium differentiation	1.855	0.815-4.22	0.140
Low differentiation	1.694	0961-5.460	0.210
Stages(distant)			
Localized	8.035	4.502-14.340	**0.000**
Regional	4.904	2.790-8.620	**0.000**
Radiotherapy(other)			
Beam radiotherapy	0.846	0.352-2.040	0.710
Brachytherapy	1.337	0.527-3.390	0.540
Combination	0.995	0.389-8.260	0.990
Radioisotopes	1.793	0.955-2.570	0.450
**Surgery**	1.415	1.140-1.760	**0.002**

The p values less than 0.05 were highlighted with bold values.

Additionally, the external test cohort was performed to verify the model. However, due to the singleness of race in the external test cohort (all cases were yellow race), this model formula could not be fitted. So, the variable “race” in formula was excluded. The calibration curve of the nomogram confirmed that the probability of predicting 5,10,15,19-year latency was consistent with the actual observation both in the external test cohort (**Figure 10C**). And the C-index and AUC of external test cohort was 0.687 and 0.688, respectively (**Figure 11C**).

## Discussions

This study established the nomograms of onset risk, cumulative incidences and latency of secondary cancer following cervical cancer patients who received radiotherapy. For the onset risk, older age, surgery, earlier stages, SCC, white and black races, were the independent risk factors of secondary cancer following cervical cancer. While the key factors that influencing latency between primary and secondary cancer onset were age, surgeries, stages and pathological types of primary cervical cancer.

Radiotherapy increased the risk of secondary cancers among cervical cancer patients and was associated with a significantly higher risk of developing a secondary cancer in the colon, rectum, anus, lung, bronchus, corpus uteri, ovary, and urinary bladder (3). Not only the SEER cohort but also the external test cohort highlighted these results. A previous meta-analysis reported an increased risk for rectal cancer after radiotherapy for cervical cancer (6). We obtained similar results in the present study. When considering all cervical cancer cases in the SEER database between 2006 and 2015, it was clearly evident that radiotherapy was an independent risk for secondary cancer. Radiotherapy kills tumor tissue by damaging DNA; however, damage could also be incurred by the adjacent organs cells at the same time. In the present study, we found that different radiotherapy methods had few impacts on the onset and latency of SPM. These results suggested that as different radiotherapies methods emerge, it will be impossible to fully avoid the risk of radiation damage.

White and black races are known to be vulnerable to SPM regardless of the type of primary cancer (7). In a previous study, Xu et al. reported that white race was an independent risk factor for SPM in patients with primary ovarian cancer (8). Similar results were subsequently reported for the risk of SPM in patients with germ cell cancers (9). We obtained similar findings in the present study; white and black races showed a higher incidence of SPM and these races had a significantly higher risk of SPM. However, this relationship remains ambiguous because of the lack of specific studies related to race and the risk of SPM. However, previous studies (7–9), suggested that this may be due to genomic differences, thus leading to differing abilities to repair radiation damage. Regrettably, the patients in external test cohort from our hospital were yellow race females, resulting in lack of evidence to assess the influences of race in SPM onset and latency period further, which needed more cases including different races to study that.

In this study, we found that advanced age was an independent risk factor for both the onset of secondary cancer and OS. A similar result was obtained in a previous multivariate analysis of thyroid cancer in that the age at first radiotherapy increased the risk of secondary cancer (10). Another study showed that the incidence of secondary cancers in patients aged >40 years at diagnosis increased with the number of treatments for hairy cell leukemia (11). In another study, Sauder et al. reported that secondary cancer was more frequent in patients who were older (12). On the one hand, with body aging, there are more carcinogenic factors and the potential for abnormal cell accumulation; this may increase the risk of secondary cancer (13). On the other hand, immunosenescence can result in faulty immune processes which provide opportunities for tumor escape (14). Based on published evidence, it is possible to speculate that an older age can increase the risk of secondary cancer and reduce the latency of SPM in patients with cervical cancer who received radiotherapy. In particular, with regard to early latency (5-10 years), an older age significantly increased the risk of SPM; this means that the older the patient, the more carcinogenic factors and greater the vulnerability of the immune system.

In addition, we found that receiving surgery for the treatment of cervical cancer was an independent risk factor for the onset of secondary cancer. Moreover, surgery reduced the risk of overall mortality. With regard to cervical cancer, patients who receive surgeries are always in the early stages of disease. This suggests that surgery is likely to be an indirect cause for secondary cancers. On the one hand, the patients who received surgery were in the early stages of disease (2). The eradication of lesions would lead to more ideal effects and result in a better prognosis and a longer lifespan. However, a longer lifespan would create more chances for the onset of secondary cancer. On the other hand, it is likely that some potential oncogenes become activated in these patients. These activated oncogenes could induce the onset of cervical cancer and trigger secondary cancer after radiotherapy (4). However, this theory remains ambiguous in that surgery increased the risk of secondary cancer in cervical cancer patients who received radiotherapy; there is a need to verify these findings in a larger cohort of patients. As a critical treatment for cervical cancer, surgery is still indispensable to promote the prognosis of early-stage cervical cancer. However, the risk of radiotherapy should be considered carefully; it is also necessary to promote radiation protection.

Interestingly, localized and regional stages, rather than distant metastatic stages, increase the risk of onset for secondary cancer. Jiang et al. constructed a nomogram to predict the prognosis of patients with cervical cancer and found that the stage of cancer was a risk factor for cervical cancer, irrespective of OS or CSS (15) Furthermore, an advanced stage was identified as an independent risk factor for death in patients with cervical cancer who receive brachytherapy (16). An advanced stage implies that cervical cancer has invaded widely and distantly, thus indicating a non-satisfactory outcome, regardless of the onset of secondary cancer. In other words, patients with a distant metastatic stage have a survival time that is too short for them to suffer from SPM. This explains the reduced risk of SPM in these patients, at least to some extent.

The pathological types of primary cervical cancer play an important role in the onset of SPM. We found that SCC was an independent risk factor for SPM; when compared to other pathological types, patients with SCC are at a greater risk from SPM due to their longer survival period. In a previous study, Gallardo-Alvarado et al. found that the recurrence rate of SCC was lower and the five-year overall survival and disease-free survival were longer when compared to ADC (17). In another study, Liu et al. reported that for the same stages, and irrespective of radiotherapy or chemotherapy, SCC resulted in better treatment effects and prognoses (18). Analysis also showed that NE accounted for the lowest risk score for the onset of SPM in our nomograms; this was due to poor survival. Overall, cervical NE was associated with a poor prognosis, despite presentation at a relatively early stage of disease (19). Furthermore, survival was lower for NE when compared to SCC for patients with cervical cancer (20). Therefore, NE and SCC were responsible for the lowest and highest scores in the nomogram, respectively.

There are some limitations in this study that need to be considered. First, the SEER data does not include detailed patient information, such as HPV infectious status. Second, due to the absence of data relating to specific radiotherapy methods, it was not possible to evaluate the effects of different radiotherapy methods on the onset of secondary cancer. Besides, the external test cohort data from our hospital was short of white and black races. Finally, we did not consider different types of secondary cancer; it is possible that different types could also influence OS. These limitations need to be investigated in future research.

## Conclusions

Standard forms of treatment for cervical cancer can improve the prognosis of patients but can also increase the incidence of SPM. In particular, an older age, surgery, earlier stages, and white and black race were identified as independent risk factors for SPM in patients with cervical cancer after radiotherapy. Although radiotherapy has been proven to be a risk factor for SPM, other features of primary cervical cancer can still exert impact on the onset latency of SPM. Therefore, it is important to carefully manage older patients for the onset of SPM in those of white and black race, those with earlier stages of disease, and those receiving surgery and radiotherapy.

## Data availability statement

The original contributions presented in the study are included in the article/**Supplementary Material**. Further inquiries can be directed to the corresponding author.

## Ethics statement

The studies involving human participants were reviewed and approved by the Ethics committee of Guangxi Medical University Cancer Hospital. Written informed consent for participation was not required for this study in accordance with the national legislation and the institutional requirements.

## Author contributions

DY acted as the guarantor of this study and conceived the idea. MC and XP analyzed the data and wrote this paper. And HW performed manuscript editing. All authors contributed to the article and approved the submitted version.
