# Supplementary material for: The risk and latency evaluation of secondary primary malignancies of cervical cancer patients who received radiotherapy: A study based on the SEER database
Source: Front Oncol. 2023 Jan 19;12:1054436. doi: 10.3389/fonc.2022.1054436 (PMC9894156; doi:10.3389/fonc.2022.1054436)
Supplement: Supplementary file 2 [file Table_2.docx]

| **Supplement 2. Patient characteristics of SEER cohort.** | | | | |
| --- | --- | --- | --- | --- |
|  | **SPM** | **Non -SPM** | **Z/X2** | **p** |
| **Age** | 53.91±12.08 | 51.64±14.40 | -4.800 | **0.000** |
| **Race** |  |  | 11.636 | **0.003** |
| Black | 80(14.26%) | 2787(14.76%) |  |  |
| White | 447(79.68%) | 14134(74.87%) |  |  |
| Other | 34(6.06%) | 1957(10.37%) |  |  |
| **Pathological types*** |  |  | 15.518 | **0.003** |
| SCC | 438(78.07%) | 13943(73.86%) |  |  |
| ADC | 94(16.75%) | 3406(18.04%) |  |  |
| ASC | 24(4.28%) | 853(4.52%) |  |  |
| NE | 1(0.18%) | 245(1.30%) |  |  |
| other | 4(0.71%) | 431(2.28%) |  |  |
| **Histological grades** |  |  | 6.102 | 0.107 |
| High differentiation | 48(8.56%) | 1382(7.32%) |  |  |
| Medium differentiation | 247(44.03%) | 7885(41.77%) |  |  |
| Low differentiation | 255(45.45%) | 8943(47.37%) |  |  |
| undifferentiation | 11(1.96%) | 668(3.54%) |  |  |
| **stages** |  |  | 60.058 | **0.000** |
| Localized | 188(33.51%) | 4563(24.17%) |  |  |
| Regional | 349(62.21%) | 11561(61.24%) |  |  |
| Distant | 24(4.28%) | 2754(14.59%) |  |  |
| **Radiotherapy** |  |  | 11.522 | **0.026** |
| Beam radiotherapy | 249(44.39%) | 9448(50.05%) |  |  |
| Brachytherapy | 45(8.02%) | 1282(6.79%) |  |  |
| Combination | 253(45.10%) | 7888(41.78%) |  |  |
| Radioisotopes | 3(0.53%) | 50(0.26%) |  |  |
| other | 11(1.96%) | 210(1.11%) |  |  |
| **surgery** |  |  | 17.989 | **0.000** |
| Yes | 294(52.41%) | 8192(43.39%) |  |  |
| no | 267(47.59%) | 10686(56.61%) |  |  |
| **chemotherapy** |  |  | 2.042 | 0.153 |
| Yes | 420(74.87%) | 14617(77.43%) |  |  |
| No | 141(25.13%) | 4261(22.57%) |  |  |
| *SCC: squamous carcinoma; ADC: adenocarcinoma; ASC: adenosquamous carcinoma; NE: neuroendocrine carcinoma | | | | |
