# Supplementary material for: The risk and latency evaluation of secondary primary malignancies of cervical cancer patients who received radiotherapy: A study based on the SEER database
Source: Front Oncol. 2023 Jan 19;12:1054436. doi: 10.3389/fonc.2022.1054436 (PMC9894156; doi:10.3389/fonc.2022.1054436)
Supplement: Supplementary file 4 [file Table_4.docx]

| **Supplement 4. Patient characteristics of external test cohort.** | | | | |
| --- | --- | --- | --- | --- |
|  | **SPM** | **Non -SPM** | **Z/X2/Fisher** | **p** |
| **Age** | 52.56±11.76 | 52.06±9.88 | -0.125 | 0.900 |
| **Pathological types** |  |  |  |  |
| SCC | 26(96.30%) | 1269(86.98%) | 3.001 | 0.779 |
| ADC | 1(3.70%) | 121(8.29%) |  |  |
| ASC | 0 | 553(3.79%) |  |  |
| NE | 0 | 15(1.03%) |  |  |
| other | 0 | 1(0.07%) |  |  |
| **Histological grades** |  |  | 1.489 | 0.438 |
| High differentiation | 18(66.67%) | 1087(74.50%) |  |  |
| Medium differentiation | 9(33.33%) | 336(23.03%) |  |  |
| Low differentiation | 0 | 36(2.47%) |  |  |
| undifferentiation | 0 | 0 |  |  |
| **stages** |  |  | 53.573 | **0.000** |
| Localized | 3(11.11%) | 291(19.95%) |  |  |
| Regional | 11(40.74%) | 1126(77.18%) |  |  |
| Distant | 13(48.15%) | 42(28.19%) |  |  |
| **Radiotherapy** |  |  | 2.438 | 0.270 |
| Beam radiotherapy | 10(37.04%) | 660(45.24%) |  |  |
| Brachytherapy | 0 | 88(6.03%) |  |  |
| Combination | 17(62.96%) | 711(48.73%) |  |  |
| Radioisotopes | 0 | 0 |  |  |
| other | 0 | 0 |  |  |
| **surgery** |  |  | 1.426 | 0.232 |
| Yes | 13(48.15%) | 539(36.94%) |  |  |
| no | 14(51.85%) | 920(64.06%) |  |  |
| **chemotherapy** |  |  | / | **0.000** |
| Yes | 25(92.59%) | 878(60.18%) |  |  |
| No | 2(7.41%) | 581(39.82%) |  |  |
